# A rare neurological complication of dengue: Guillain-Barré Syndrome in a dengue fever patient

**DOI:** 10.1016/j.idcr.2025.e02160

**Published:** 2025-01-16

**Authors:** Ibrahim Nagmeldin Hassan, Siddig Yaqub, Mohamed Ibrahim, Ghada Aljaili, Nagmeldin Abuassa

**Affiliations:** a University of Khartoum, Faculty of Medicine, Khartoum, Sudan; b Karary University, Faculty of Medicine, Khartoum, Sudan; c Al-Neelain University Faculty of Medicine, Khartoum, Sudan; d Omdurman Islamic University, Faculty of Medicine, Khartoum, Sudan

**Keywords:** Dengue fever, Guillain-Barré Syndrome, Acute flaccid paralysis, Intravenous immunoglobulin (IVIG), Neurological complications

## Abstract

**Background:**

Dengue fever is a common viral illness in tropical and subtropical regions, typically presenting with fever, myalgia, and hemorrhagic symptoms. While neurological complications are rare, Guillain-Barré Syndrome (GBS) is a known but uncommon sequelae of viral infections, including dengue.

**Case presentation:**

We report the case of a previously healthy 32-year-old male who developed acute flaccid paralysis secondary to GBS following a dengue fever infection. The patient initially presented with classic dengue symptoms—fever, severe headache, retro-orbital pain, and myalgia. Following resolution of the acute dengue phase, he developed ascending muscle weakness, areflexia, and numbness in both legs. Electromyography confirmed a diagnosis of GBS, and cerebrospinal fluid analysis revealed albuminocytologic dissociation. The patient was treated with intravenous immunoglobulin (IVIG), leading to significant clinical improvement, with gradual recovery of motor function.

**Discussion:**

This case highlights the rare but significant neurological complication of GBS following dengue fever. Clinicians should maintain a high index of suspicion for GBS in patients recovering from dengue, especially in endemic regions. Early diagnosis and treatment with IVIG or plasmapheresis are critical for improving outcomes in these patients.

**Conclusion:**

Dengue fever can lead to severe neurological sequelae such as GBS, and early recognition and intervention can prevent long-term disability. This case underscores the need for vigilance in identifying such complications in patients recovering from viral infections.

## Highlights

• Rare case of Guillain-Barré Syndrome after dengue fever highlights the need for awareness in endemic regions.
• Recognizing albuminocytologic dissociation in CSF is crucial for GBS diagnosis.
• IVIG therapy shows positive outcomes for GBS recovery post-dengue infection.

## Introduction

Dengue fever, caused by the dengue virus (DENV), is a common infection in tropical and subtropical regions, where it causes periodic outbreaks. The clinical spectrum of dengue fever can vary from asymptomatic cases to severe manifestations, such as dengue hemorrhagic fever and dengue shock syndrome [1]. Although neurological complications of dengue are rare, they have been documented, including encephalitis, transverse myelitis, and Guillain-Barré Syndrome (GBS) [2].

GBS is an autoimmune disorder characterized by an acute onset of ascending weakness, sensory disturbances, and loss of reflexes, commonly occurring after viral infections [3]. While post-infectious GBS is well known to be triggered by viruses like Zika and Epstein-Barr virus, its association with dengue fever is rare and less well-understood [4].

In this case report, we discuss a patient who developed GBS following a confirmed dengue fever infection.

## Case presentation

A 32-year-old previously healthy male presented to the emergency department with a 5-day history of high-grade fever, myalgia, and generalized fatigue. He reported that the fever was persistent, often exceeding 39°C, and was associated with chills and rigors. He also experienced severe muscle pain, particularly in the lower back and legs, which significantly limited his mobility. On initial evaluation, the patient was alert but appeared uncomfortable due to pain. Vital signs revealed a heart rate of 110 beats per minute, blood pressure of 100/60 mmHg, and a temperature of 38.5°C. The physical examination noted mild hepatomegaly, but there were no signs of a rash, lymphadenopathy, or jaundice. No neurological deficits were observed at this stage.

Initial laboratory investigations were conducted, revealing thrombocytopenia with a platelet count of 85,000/mm³ (normal range: 150,000–450,000/mm³) and mild elevations in liver enzymes, specifically aspartate aminotransferase (AST) at 75 U/L and alanine aminotransferase (ALT) at 85 U/L (normal range: 10–40 U/L). Hematocrit levels were stable at 44 %, and a complete blood count showed leukopenia with a white blood cell count of 4000/mm³ . Serological testing for dengue fever was conducted, resulting in positive IgM and IgG antibodies, confirming a diagnosis of dengue fever. The patient was treated conservatively with supportive care, including intravenous hydration and acetaminophen for fever management. After three days of treatment, his fever resolved, and he experienced a gradual improvement in symptoms. He was discharged from the hospital on day 8 with advice to monitor for any new or worsening symptoms.

On day 10 after the onset of fever, the patient returned to the emergency department, reporting the sudden onset of weakness that began in his legs. He described the weakness as starting distally in the feet and progressing rapidly upwards, with noticeable difficulty in walking. Within 24 hours, he developed weakness in his arms, rendering him unable to lift his arms above shoulder level. Additionally, he reported paresthesia, including tingling and a “pins and needles” sensation in both hands and feet. During the neurological examination, the following findings were noted: bilateral, symmetric flaccid paralysis of all four limbs, with the patient being unable to perform movements against gravity. Deep tendon reflexes were absent, with no signs of muscle atrophy. Sensory examination revealed a diminished response to light touch and pinprick sensations in a glove-and-stocking distribution. Cranial nerves were intact, and there was no evidence of respiratory distress or bulbar involvement.

Given the rapid progression of neurological symptoms, the patient was admitted to the intensive care unit (ICU) for close monitoring and further evaluation. An urgent electromyography (EMG) and nerve conduction study were performed, which revealed significant demyelination characterized by prolonged distal motor latencies and reduced conduction velocities, confirming the diagnosis of acute inflammatory demyelinating polyneuropathy (AIDP), the most common subtype of Guillain-Barré Syndrome. A lumbar puncture was also performed to analyze cerebrospinal fluid (CSF). The CSF analysis revealed an elevated protein level of 110 mg/dL (normal range: 15–45 mg/dL) with a normal white blood cell count, consistent with albuminocytologic dissociation—a hallmark of GBS.

Given the diagnosis of Guillain-Barré Syndrome secondary to dengue infection, the patient was started on intravenous immunoglobulin (IVIG) therapy at a dose of 0.4 g/kg/day for five days. Additionally, early physical therapy was initiated to prevent complications related to immobility and to aid in recovery. Throughout his ICU stay, the patient was closely monitored for respiratory function, as GBS can lead to respiratory muscle weakness. Fortunately, he did not exhibit any respiratory compromise. Over the course of treatment, the patient began to show signs of improvement in muscle strength and sensation, with gradual recovery noted in both upper and lower extremities.

By the end of his hospitalization, approximately four weeks post-admission, he had regained near-complete strength in his upper limbs. However, he continued to experience mild residual weakness in his lower limbs, particularly with activities requiring strength in the hip flexors and ankle dorsiflexors. He was discharged from the hospital with a comprehensive rehabilitation plan and outpatient follow-up. At his three-month follow-up visit, the patient reported significant recovery, with nearly full strength regained in all extremities and the ability to walk independently without assistance. He was encouraged to continue his outpatient physical therapy to maximize recovery and prevent long-term complications.

## Discussion

This case illustrates a rare but significant complication of dengue fever: Guillain-Barré Syndrome (GBS). While dengue fever is primarily known for causing high fever, myalgia, and hemorrhagic manifestations, neurological complications are less common yet critical to recognize. GBS is an acute inflammatory demyelinating polyneuropathy that often follows a variety of infectious agents, with viral infections being among the most common triggers. The pathophysiology of GBS is thought to involve molecular mimicry, where antibodies generated in response to a viral infection mistakenly attack peripheral nerve myelin, leading to demyelination and subsequent motor weakness [3], [5].

The link between dengue fever and GBS has been documented in a limited number of studies. In a cohort study of dengue patients, the incidence of GBS was found to be approximately 9.26 % among those with dengue fever, although this may vary based on geographical region and local disease prevalence [6]. The mechanisms by which dengue virus induces GBS are not entirely understood, but the involvement of anti-dengue antibodies that cross-react with peripheral nerve antigens has been proposed as a potential explanation [5].

In this case, the patient presented with classic symptoms of dengue fever followed by the sudden onset of GBS, which aligns with reported cases in the literature. Similar cases have documented the temporal relationship between dengue infection and the onset of GBS, often occurring within days to weeks following the resolution of fever [7]. This emphasizes the importance of maintaining a high index of suspicion for GBS in patients recovering from dengue, especially in endemic areas.

The diagnosis of GBS relies on clinical findings, supported by electrophysiological studies and CSF analysis. Electromyography and nerve conduction studies typically reveal demyelinating features, such as reduced conduction velocities and prolonged latencies, which were observed in our patient. Additionally, the finding of albuminocytologic dissociation in the CSF analysis, with elevated protein and normal white blood cell count, corroborated the diagnosis of GBS [5], [6]. Recognizing these features is crucial for prompt intervention, as early treatment with intravenous immunoglobulin (IVIG) or plasmapheresis has been shown to improve outcomes and reduce the duration of disability [8].

Management of GBS remains supportive, with the primary focus on maintaining respiratory function and mobilizing patients as soon as feasible to prevent complications associated with immobility. This case highlights the importance of interdisciplinary care, involving neurologists, intensivists, and rehabilitation specialists to optimize recovery. Our patient’s successful recovery following IVIG treatment underscores the efficacy of early therapeutic intervention in mitigating the effects of GBS.

In conclusion, this case serves as a reminder for healthcare providers in dengue-endemic regions to be vigilant for neurological symptoms in patients recovering from dengue fever. The prompt recognition of GBS can facilitate timely treatment and improve clinical outcomes. Given the potential for GBS to cause significant morbidity, further studies are needed to explore the relationship between dengue virus infection and the risk of developing GBS, as well as to better understand the underlying mechanisms involved.

## Ethical approval

Written informed consent was obtained from the patient for publication. A copy of the written consent is available for review by the Editor-in-Chief of this journal on request.

## Author agreement statement

We the undersigned declare that this manuscript is original, has not been published before and is not currently being considered for publication elsewhere.

We confirm that the manuscript has been read and approved by all named authors and that there are no other persons who satisfied the criteria for authorship but are not listed. We further confirm that the order of authors listed in the manuscript has been approved by all of us.

We understand that the Corresponding Author is the sole contact for the Editorial process. He/she is responsible for communicating with the other authors about progress, submissions of revisions and final approval of proofs

## Author contributions

All authors read and approved the final manuscript.

## Funding

None declared.

## CRediT authorship contribution statement

**Ibrahim Nagmeldin Hassan:** Conceptualization, Project administration, Supervision, Validation, Visualization, Writing – original draft, Writing – review & editing. **Mohamed Ibrahim:** Conceptualization, Writing – original draft, Writing – review & editing. **Ghada Aljaili:** Supervision, Writing – review & editing. **Nagmeldin Abuassa:** Writing – original draft, Writing – review & editing. **Siddig Yaqub:** Writing – original draft, Writing – review & editing.

## Declaration of Competing Interest

The authors declare that they have no known competing financial interests or personal relationships that could have appeared to influence the work reported in this paper.

## References

[1] Khan M.B., Yang Z.S., Lin C.Y., Hsu M.C., Urbina A.N., Assavalapsakul W. (2023 Oct). Dengue overview: an updated systemic review. J Infect Public Health.

[2] Trivedi S., Chakravarty A. (2022 Aug). Neurological complications of dengue fever. Curr Neurol Neurosci Rep.

[3] Bellanti R., Rinaldi S. (2024 Aug). Guillain-Barré syndrome: a comprehensive review. Eur J Neurol.

[4] Dalugama C., Shelton J., Ekanayake M., Gawarammana I.B. (2018 May 15). Dengue fever complicated with Guillain-Barré syndrome: a case report and review of the literature. J Med Case Rep.

[5] van den Berg B., Walgaard C., Drenthen J., Fokke C., Jacobs B.C., van Doorn P.A. (2014 Aug). Guillain-Barré syndrome: pathogenesis, diagnosis, treatment and prognosis. Nat Rev Neurol.

[6] Sahu R., Verma R., Jain A., Garg R.K., Singh M.K., Malhotra H.S. (2014 Oct 28). Neurologic complications in dengue virus infection: a prospective cohort study. Neurology.

[7] Ralapanawa D.M., Kularatne S.A., Jayalath W.A. (2015 Nov 27). Guillain-Barre syndrome following dengue fever and literature review. BMC Res Notes.

[8] Hauser S.L., Asbury A.K. (2005). Guillain-Barré syndrome and other immune-mediated neuropathies. Harrisons Princ Intern Med.

